# 1,3-Phenyl­ene bis­{3-[2-chloro-4-(tri­fluoro­meth­yl)phen­oxy]benzoate}

**DOI:** 10.1107/S1600536812030826

**Published:** 2012-07-14

**Authors:** Hao Peng, Hongwu He

**Affiliations:** a Key Laboratory of Pesticide and Chemical Biology, College of Chemistry, Central China Normal University, Wuhan 430079, People’s Republic of China

## Abstract

In the title compound, C_34_H_18_Cl_2_F_6_O_6_, one terminal trifluoro­methyl and one entire 2-chloro-4-(trifluoro­meth­yl)phenyl group are disordered with refined occupancy ratios of 0.715 (11):0.285 (11) and 0.517 (5):0.429 (5), respectively. In the crystal, weak inter­molecular C—H⋯O hydrogen bonds link the mol­ecules into ribbons propagating along the *a*-axis direction.

## Related literature

For the herbicidal activity of phen­oxy­phenyl derivatives, see: Dayan & Allen (2000 ▶). For the structures of related compounds, see: Peng & He (2006 ▶, 2007 ▶). For standard bond-length data, see: Allen *et al.* (1987 ▶).
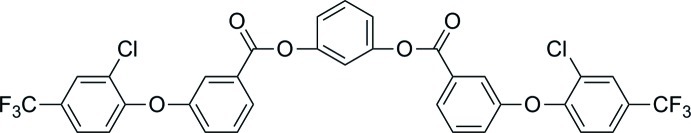


## Experimental

### 

#### Crystal data

C_34_H_18_Cl_2_F_6_O_6_
*M* *_r_* = 707.38
Triclinic, 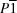

*a* = 7.7175 (11) Å
*b* = 8.7399 (12) Å
*c* = 23.973 (3) Å
α = 92.986 (2)°
β = 98.485 (3)°
γ = 92.611 (3)°
*V* = 1594.8 (4) Å^3^
*Z* = 2
Mo *K*α radiation
μ = 0.28 mm^−1^
*T* = 292 K
0.30 × 0.20 × 0.20 mm

#### Data collection

Bruker SMART APEX CCD area-detector diffractometer
13550 measured reflections
5564 independent reflections
3199 reflections with *I* > 2σ(*I*)
*R*_int_ = 0.062

#### Refinement

*R*[*F* ^2^ > 2σ(*F* ^2^)] = 0.079
*wR*(*F* ^2^) = 0.257
*S* = 1.00
5564 reflections
528 parameters
215 restraints
H-atom parameters constrained
Δρ_max_ = 0.51 e Å^−3^
Δρ_min_ = −0.36 e Å^−3^

### 

Data collection: *SMART* (Bruker, 2001 ▶); cell refinement: *SAINT-Plus* (Bruker, 2001 ▶); data reduction: *SAINT-Plus*; program(s) used to solve structure: *SHELXS97* (Sheldrick, 2008 ▶); program(s) used to refine structure: *SHELXL97* (Sheldrick, 2008 ▶); molecular graphics: *PLATON* (Spek, 2009 ▶); software used to prepare material for publication: *PLATON*.

## Supplementary Material

Crystal structure: contains datablock(s) global, I. DOI: 10.1107/S1600536812030826/bg2468sup1.cif

Structure factors: contains datablock(s) I. DOI: 10.1107/S1600536812030826/bg2468Isup2.hkl

Supplementary material file. DOI: 10.1107/S1600536812030826/bg2468Isup3.cml

Additional supplementary materials:  crystallographic information; 3D view; checkCIF report

## Figures and Tables

**Table 1:** Hydrogen-bond geometry (Å, °)

*D*—H⋯*A*	*D*—H	H⋯*A*	*D*⋯*A*	*D*—H⋯*A*
C20—H20⋯O1^i^	0.93	2.50	3.412 (5)	166
C10—H10⋯O2^ii^	0.93	2.59	3.152 (5)	120

Symmetry codes: (i) 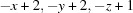; (ii) 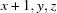.

**Fig. 1.**
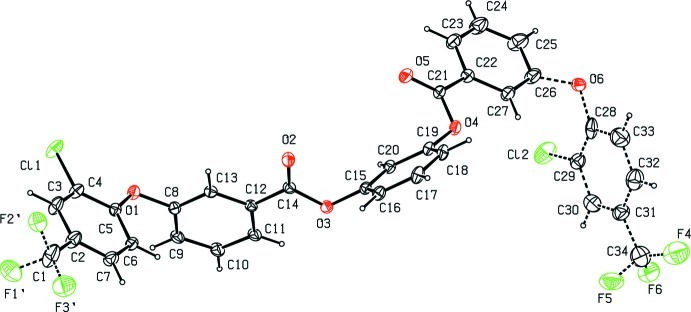
Molecular structure of the title compound, with 50% probability displacement ellipsoids. Disordered parts are represented by their major components, and drawn in broken lines.

## supplementary crystallographic information

### Comment

Phenoxyphenyl derivatives exhibit herbicidal activity by interacting with plant metabolism in multiple ways. Many diphenyl ether inhibitors of protoporphyrinogen oxidase contains 2-chloro-4-(trifluoromethyl)phenoxy benzoate moiety, which have been a successful class of herbicides (Dayan & Allen, 2000). The title compound has been prepared as a part our work on the design and synthesis of novel herbicidal compounds.

Fig. 1 shows the crystal structure of the title compound. Bond lengths and angles show normal values (Allen *et al.*, 1987). The terminal trifluoromethyl and one of 2-chloro-4-(trifluoromethyl)phenoxy groups are disordered and required a strongly restrained refinement. The C28···C33 ring, *Cg*5, is rotated almost 180° to the C28'···C33' ring, *Cg*6, by dihedral angle 2.79°. In the crystal, molecules are linked *via* weak intermolecular C—H···O hydrogen bonds (Table 1).

### Experimental

3-(2-Chloro-4-(trifluoromethyl)phenoxy)benzoyl chloride (0.005 mol) in chloroform was added dropwise at 275–278 K to a stirred solution of phen-1,3-diol (0.0025 mol) and triethylamine (0.005 mol) in chloroform (25 mL). The mixture was stirred at 275–278 K for 1 h, washed with 1% hydrochloric acid solution, followed by sodium hydrogen carbonate and ice water, dried and evaporated. The residue was purified by chromatography (silica gel with 15% acetone in petroleum ether). Recrystallization from ethyl acetate and petroleum ether over 1 week gave colorless blocks of the title compound.

### Refinement

The trifluoromethyl group appeared disordered over two orientations with refined occupancies of 0.715 (11) and 0.285 (11) for the major and minor components, respectively. The distances between six pairs of atoms (F1—F2, F1—F3, F2—F3, F1'-F2', F1'-F3', and F2'-F3') were restrained to be equal with the standard deviation (0.01). A similar split refinement was applied to a disordered 2-chloro-4-(trifluoromethyl)phenoxy group, leading to occupation factors of 0.571 (5), 0.429 (5). The displacement parameters of the disordered atoms were restrained to approximately isotropic behavior. H atoms were geometrically positioned (C*sp*^2^—H = 0.93 Å, Cmethine—H = 0.98 Å, Cmethylene—H = 0.97 Å, Cmethyl—H = 0.96 Å) and refined as riding, with *U*_iso_(H) = *xU*_eq_(C), where *x* = 1.5 for methyl H and 1.2 for all other H atoms.

### Crystal data

**Table:**

C_34_H_18_Cl_2_F_6_O_6_	*Z* = 2
*M**_r_* = 707.38	*F*(000) = 716
Triclinic, *P*1	*D*_x_ = 1.473 Mg m^−^^3^
*a* = 7.7175 (11) Å	Mo *K*α radiation, λ = 0.71073 Å
*b* = 8.7399 (12) Å	Cell parameters from 2828 reflections
*c* = 23.973 (3) Å	θ = 2.3–23.0°
α = 92.986 (2)°	µ = 0.28 mm^−^^1^
β = 98.485 (3)°	*T* = 292 K
γ = 92.611 (3)°	Block, yellow
*V* = 1594.8 (4) Å^3^	0.30 × 0.20 × 0.20 mm

### Data collection

**Table:**

Bruker SMART APEX CCD area-detector diffractometer	3199 reflections with *I* > 2σ(*I*)
Radiation source: fine-focus sealed tube	*R*_int_ = 0.062
Graphite monochromator	θ_max_ = 25.0°, θ_min_ = 1.7°
φ and ω scans	*h* = −9→9
13550 measured reflections	*k* = −10→10
5564 independent reflections	*l* = −25→28

### Refinement

**Table:**

Refinement on *F*^2^	Primary atom site location: structure-invariant direct methods
Least-squares matrix: full	Secondary atom site location: difference Fourier map
*R*[*F*^2^ > 2σ(*F*^2^)] = 0.079	Hydrogen site location: inferred from neighbouring sites
*wR*(*F*^2^) = 0.257	H-atom parameters constrained
*S* = 1.00	*w* = 1/[σ^2^(*F*_o_^2^) + (0.154*P*)^2^] where *P* = (*F*_o_^2^ + 2*F*_c_^2^)/3
5564 reflections	(Δ/σ)_max_ < 0.001
528 parameters	Δρ_max_ = 0.51 e Å^−^^3^
215 restraints	Δρ_min_ = −0.36 e Å^−^^3^

### Special details

**Table:**

Geometry. All e.s.d.'s (except the e.s.d. in the dihedral angle between two l.s. planes) are estimated using the full covariance matrix. The cell e.s.d.'s are taken into account individually in the estimation of e.s.d.'s in distances, angles and torsion angles; correlations between e.s.d.'s in cell parameters are only used when they are defined by crystal symmetry. An approximate (isotropic) treatment of cell e.s.d.'s is used for estimating e.s.d.'s involving l.s. planes.
Refinement. Refinement of *F*^2^ against ALL reflections. The weighted *R*-factor *wR* and goodness of fit *S* are based on *F*^2^, conventional *R*-factors *R* are based on *F*, with *F* set to zero for negative *F*^2^. The threshold expression of *F*^2^ > σ(*F*^2^) is used only for calculating *R*-factors(gt) *etc*. and is not relevant to the choice of reflections for refinement. *R*-factors based on *F*^2^ are statistically about twice as large as those based on *F*, and *R*- factors based on ALL data will be even larger.

### Fractional atomic coordinates and isotropic or equivalent isotropic displacement parameters (Å^2^)

**Table:**

	*x*	*y*	*z*	*U*_iso_*/*U*_eq_	Occ. (<1)
C1	1.0008 (10)	0.4068 (9)	0.1855 (3)	0.164 (4)	
F1	1.1346 (11)	0.3142 (8)	0.1888 (3)	0.173 (3)	0.715 (11)
F2	0.9704 (16)	0.4550 (9)	0.1344 (2)	0.181 (4)	0.715 (11)
F3	0.8624 (10)	0.3082 (8)	0.1916 (3)	0.178 (3)	0.715 (11)
F1'	1.1403 (17)	0.434 (2)	0.1557 (7)	0.172 (8)	0.285 (11)
F2'	0.8633 (17)	0.4334 (18)	0.1450 (6)	0.129 (6)	0.285 (11)
F3'	0.997 (3)	0.2580 (12)	0.1905 (9)	0.189 (9)	0.285 (11)
C2	1.0228 (9)	0.5297 (6)	0.2317 (2)	0.1074 (18)	
C3	1.0153 (8)	0.6824 (6)	0.2186 (2)	0.1061 (17)	
H3	0.9965	0.7081	0.1811	0.127*	
C4	1.0356 (6)	0.7936 (5)	0.26078 (19)	0.0780 (12)	
C5	1.0635 (5)	0.7577 (4)	0.31719 (16)	0.0606 (9)	
C6	1.0725 (6)	0.6045 (5)	0.32885 (18)	0.0718 (11)	
H6	1.0915	0.5779	0.3662	0.086*	
C7	1.0540 (7)	0.4930 (6)	0.2868 (2)	0.0921 (14)	
H7	1.0627	0.3908	0.2955	0.111*	
Cl1	1.0268 (2)	0.98315 (14)	0.24459 (6)	0.1118 (6)	
C8	1.0931 (5)	0.8438 (4)	0.41370 (16)	0.0633 (10)	
C9	1.2532 (5)	0.8562 (5)	0.44654 (19)	0.0730 (11)	
H9	1.3538	0.8795	0.4309	0.088*	
C10	1.2640 (5)	0.8340 (6)	0.50298 (19)	0.0803 (13)	
H10	1.3727	0.8449	0.5258	0.096*	
C11	1.1163 (5)	0.7958 (5)	0.52665 (17)	0.0727 (12)	
H11	1.1251	0.7795	0.5650	0.087*	
C12	0.9547 (4)	0.7821 (4)	0.49233 (15)	0.0568 (9)	
C13	0.9418 (5)	0.8086 (4)	0.43542 (16)	0.0583 (9)	
H13	0.8333	0.8028	0.4124	0.070*	
C14	0.7901 (5)	0.7436 (4)	0.51447 (16)	0.0605 (10)	
C15	0.6718 (5)	0.6869 (4)	0.59670 (15)	0.0593 (9)	
C16	0.5571 (6)	0.5592 (5)	0.58476 (17)	0.0718 (11)	
H16	0.5728	0.4834	0.5575	0.086*	
C17	0.4200 (7)	0.5491 (5)	0.6147 (2)	0.0841 (13)	
H17	0.3414	0.4641	0.6074	0.101*	
C18	0.3932 (6)	0.6588 (5)	0.65491 (18)	0.0762 (12)	
H18	0.2976	0.6493	0.6742	0.091*	
C19	0.5100 (5)	0.7820 (5)	0.66599 (16)	0.0659 (10)	
C20	0.6535 (5)	0.7982 (5)	0.63710 (15)	0.0629 (10)	
H20	0.7338	0.8819	0.6451	0.075*	
C21	0.4647 (5)	1.0363 (5)	0.69507 (19)	0.0743 (12)	
C22	0.4654 (6)	1.1432 (5)	0.74417 (19)	0.0790 (12)	
C23	0.4556 (8)	1.2993 (6)	0.7364 (2)	0.1024 (16)	
H23	0.4483	1.3340	0.7002	0.123*	
C24	0.4564 (11)	1.4006 (7)	0.7804 (3)	0.135 (2)	
H24	0.4531	1.5047	0.7744	0.162*	
C25	0.4619 (11)	1.3539 (8)	0.8330 (3)	0.146 (3)	
H25	0.4607	1.4248	0.8632	0.175*	
C26	0.4692 (10)	1.1990 (7)	0.8417 (2)	0.121 (2)	
C27	0.4746 (7)	1.0952 (6)	0.7987 (2)	0.0950 (15)	
H27	0.4843	0.9919	0.8055	0.114*	
O1	1.0799 (4)	0.8770 (3)	0.35637 (11)	0.0716 (8)	
O2	0.6463 (3)	0.7448 (4)	0.48806 (11)	0.0797 (9)	
O3	0.8193 (3)	0.7046 (3)	0.56895 (10)	0.0678 (8)	
O4	0.4900 (4)	0.8907 (3)	0.70907 (11)	0.0729 (8)	
O5	0.4436 (5)	1.0719 (4)	0.64716 (14)	0.1047 (11)	
C28	0.4973 (19)	1.0073 (12)	0.9132 (8)	0.114 (8)	0.429 (5)
C29	0.6790 (19)	1.0061 (12)	0.9252 (7)	0.092 (4)	0.429 (5)
C30	0.7572 (13)	0.8795 (14)	0.9487 (8)	0.116 (6)	0.429 (5)
H30	0.8788	0.8787	0.9567	0.139*	0.429 (5)
C31	0.6537 (14)	0.7541 (14)	0.9603 (10)	0.121 (3)	0.429 (5)
C32	0.4720 (14)	0.7553 (15)	0.9483 (10)	0.146 (8)	0.429 (5)
H32	0.4028	0.6714	0.9560	0.175*	0.429 (5)
C33	0.3938 (14)	0.8819 (16)	0.9247 (8)	0.160 (11)	0.429 (5)
H33	0.2722	0.8827	0.9167	0.192*	0.429 (5)
Cl2	0.8236 (8)	1.1645 (6)	0.9180 (2)	0.171 (2)	0.429 (5)
C34	0.7402 (18)	0.6295 (15)	0.9921 (6)	0.176 (4)	0.429 (5)
F4	0.6176 (18)	0.5555 (19)	1.0149 (8)	0.252 (5)	0.429 (5)
F5	0.802 (2)	0.5392 (18)	0.9542 (6)	0.200 (6)	0.429 (5)
F6	0.8722 (19)	0.6868 (17)	1.0316 (7)	0.207 (7)	0.429 (5)
O6	0.4227 (13)	1.1471 (13)	0.8941 (3)	0.083 (3)	0.429 (5)
C28'	0.5648 (16)	1.0452 (12)	0.9143 (6)	0.099 (5)	0.571 (5)
C29'	0.4545 (11)	0.9259 (14)	0.9270 (5)	0.104 (4)	0.571 (5)
C30'	0.5250 (10)	0.7945 (13)	0.9492 (6)	0.122 (4)	0.571 (5)
H30'	0.4511	0.7148	0.9577	0.146*	0.571 (5)
C31'	0.7057 (10)	0.7824 (13)	0.9587 (7)	0.121 (3)	0.571 (5)
C32'	0.8161 (10)	0.9016 (14)	0.9460 (8)	0.172 (8)	0.571 (5)
H32'	0.9370	0.8935	0.9523	0.207*	0.571 (5)
C33'	0.7456 (15)	1.0330 (12)	0.9238 (7)	0.153 (7)	0.571 (5)
H33'	0.8194	1.1128	0.9153	0.184*	0.571 (5)
Cl2'	0.2398 (7)	0.9546 (9)	0.9143 (3)	0.273 (4)	0.571 (5)
C34'	0.7793 (14)	0.6370 (13)	0.9821 (4)	0.176 (4)	0.571 (5)
F4'	0.6955 (18)	0.5089 (17)	0.9552 (5)	0.252 (5)	0.571 (5)
F5'	0.9484 (12)	0.6468 (13)	0.9771 (4)	0.207 (4)	0.571 (5)
F6'	0.7642 (15)	0.6294 (11)	1.0369 (3)	0.159 (3)	0.571 (5)
O6'	0.522 (2)	1.1809 (14)	0.9002 (3)	0.150 (4)	0.571 (5)

### Atomic displacement parameters (Å^2^)

**Table:**

	*U*^11^	*U*^22^	*U*^33^	*U*^12^	*U*^13^	*U*^23^
C1	0.272 (12)	0.130 (7)	0.086 (5)	−0.012 (8)	0.017 (6)	0.017 (5)
F1	0.256 (7)	0.111 (5)	0.157 (6)	0.041 (5)	0.058 (5)	−0.039 (4)
F2	0.296 (10)	0.152 (5)	0.091 (4)	0.017 (7)	0.019 (5)	−0.012 (3)
F3	0.235 (7)	0.127 (5)	0.150 (5)	−0.012 (5)	−0.011 (5)	−0.052 (4)
F1'	0.178 (11)	0.156 (11)	0.176 (12)	0.002 (9)	0.037 (9)	−0.054 (9)
F2'	0.147 (9)	0.120 (9)	0.116 (10)	−0.006 (7)	0.017 (7)	−0.027 (7)
F3'	0.200 (13)	0.164 (12)	0.197 (13)	0.012 (10)	0.011 (10)	0.003 (9)
C2	0.184 (6)	0.074 (3)	0.061 (3)	0.015 (3)	0.005 (3)	−0.001 (2)
C3	0.168 (5)	0.090 (4)	0.054 (3)	−0.003 (3)	0.000 (3)	0.010 (3)
C4	0.094 (3)	0.063 (2)	0.072 (3)	−0.004 (2)	−0.005 (2)	0.014 (2)
C5	0.058 (2)	0.065 (2)	0.058 (2)	−0.0022 (17)	0.0071 (17)	0.0061 (19)
C6	0.086 (3)	0.072 (3)	0.058 (2)	0.013 (2)	0.008 (2)	0.012 (2)
C7	0.130 (4)	0.070 (3)	0.078 (3)	0.020 (3)	0.013 (3)	0.006 (2)
Cl1	0.1702 (14)	0.0692 (8)	0.0854 (9)	−0.0165 (8)	−0.0159 (8)	0.0242 (6)
C8	0.068 (3)	0.065 (2)	0.058 (2)	0.0083 (18)	0.0104 (19)	0.0010 (18)
C9	0.052 (2)	0.088 (3)	0.081 (3)	0.009 (2)	0.014 (2)	0.002 (2)
C10	0.051 (2)	0.117 (4)	0.071 (3)	0.014 (2)	0.000 (2)	0.002 (3)
C11	0.055 (2)	0.102 (3)	0.061 (3)	0.020 (2)	0.0035 (19)	0.001 (2)
C12	0.051 (2)	0.063 (2)	0.054 (2)	0.0072 (16)	0.0043 (16)	−0.0042 (17)
C13	0.053 (2)	0.060 (2)	0.059 (2)	0.0023 (16)	0.0006 (17)	−0.0021 (17)
C14	0.055 (2)	0.071 (2)	0.054 (2)	0.0106 (17)	0.0024 (18)	−0.0021 (18)
C15	0.062 (2)	0.067 (2)	0.049 (2)	0.0043 (18)	0.0061 (17)	0.0102 (18)
C16	0.086 (3)	0.069 (3)	0.057 (2)	−0.003 (2)	0.003 (2)	0.0038 (19)
C17	0.098 (3)	0.082 (3)	0.068 (3)	−0.030 (3)	0.006 (3)	0.013 (2)
C18	0.081 (3)	0.083 (3)	0.067 (3)	−0.014 (2)	0.018 (2)	0.021 (2)
C19	0.076 (3)	0.074 (3)	0.048 (2)	0.004 (2)	0.0071 (19)	0.0123 (19)
C20	0.060 (2)	0.071 (2)	0.056 (2)	−0.0042 (18)	0.0027 (18)	0.012 (2)
C21	0.073 (3)	0.095 (3)	0.060 (3)	0.017 (2)	0.012 (2)	0.025 (2)
C22	0.088 (3)	0.083 (3)	0.069 (3)	0.018 (2)	0.014 (2)	0.013 (2)
C23	0.140 (5)	0.086 (4)	0.084 (4)	0.023 (3)	0.017 (3)	0.022 (3)
C24	0.213 (8)	0.079 (4)	0.111 (5)	0.031 (4)	0.013 (5)	0.010 (4)
C25	0.225 (8)	0.098 (5)	0.106 (5)	0.044 (5)	−0.009 (5)	−0.011 (4)
C26	0.197 (7)	0.100 (4)	0.070 (4)	0.055 (4)	0.013 (4)	0.011 (3)
C27	0.144 (4)	0.080 (3)	0.067 (3)	0.032 (3)	0.022 (3)	0.014 (2)
O1	0.0889 (19)	0.0666 (16)	0.0598 (17)	−0.0009 (14)	0.0135 (14)	0.0058 (13)
O2	0.0510 (16)	0.125 (3)	0.0620 (17)	0.0010 (15)	0.0011 (13)	0.0167 (16)
O3	0.0578 (15)	0.0908 (19)	0.0541 (16)	0.0127 (13)	0.0022 (12)	0.0080 (13)
O4	0.095 (2)	0.0740 (18)	0.0524 (16)	0.0045 (15)	0.0167 (13)	0.0099 (14)
O5	0.138 (3)	0.116 (3)	0.068 (2)	0.047 (2)	0.0216 (19)	0.0280 (19)
C28	0.176 (18)	0.107 (16)	0.063 (12)	−0.023 (16)	0.049 (12)	−0.009 (11)
C29	0.112 (13)	0.085 (11)	0.075 (10)	0.002 (9)	0.005 (9)	0.001 (8)
C30	0.097 (10)	0.147 (18)	0.096 (12)	0.010 (11)	−0.001 (8)	−0.019 (12)
C31	0.149 (7)	0.126 (6)	0.087 (4)	0.041 (5)	0.007 (5)	0.011 (4)
C32	0.177 (12)	0.128 (11)	0.134 (12)	−0.016 (9)	0.035 (9)	0.006 (9)
C33	0.162 (14)	0.169 (14)	0.153 (14)	0.030 (10)	0.031 (10)	−0.001 (10)
Cl2	0.192 (5)	0.132 (4)	0.175 (5)	−0.039 (3)	−0.012 (4)	0.035 (3)
C34	0.197 (7)	0.179 (7)	0.154 (7)	0.029 (6)	0.035 (6)	0.000 (6)
F4	0.278 (8)	0.222 (8)	0.255 (8)	0.045 (6)	0.017 (6)	0.031 (6)
F5	0.231 (11)	0.154 (8)	0.217 (10)	0.085 (8)	0.024 (8)	0.002 (7)
F6	0.190 (10)	0.225 (11)	0.214 (11)	0.042 (8)	0.035 (8)	0.062 (9)
O6	0.102 (6)	0.090 (6)	0.056 (5)	0.034 (5)	0.003 (4)	−0.011 (4)
C28'	0.155 (15)	0.084 (8)	0.056 (7)	0.045 (10)	−0.002 (8)	0.001 (6)
C29'	0.149 (12)	0.115 (10)	0.044 (6)	0.045 (10)	0.000 (6)	−0.008 (6)
C30'	0.160 (9)	0.123 (8)	0.086 (7)	−0.003 (8)	0.036 (7)	0.001 (6)
C31'	0.149 (7)	0.126 (6)	0.087 (4)	0.041 (5)	0.007 (5)	0.011 (4)
C32'	0.161 (10)	0.191 (12)	0.158 (12)	0.036 (9)	−0.001 (9)	−0.005 (9)
C33'	0.166 (11)	0.153 (12)	0.139 (10)	0.003 (9)	0.027 (9)	−0.009 (9)
Cl2'	0.170 (4)	0.396 (10)	0.220 (6)	0.051 (5)	−0.027 (4)	−0.156 (6)
C34'	0.197 (7)	0.179 (7)	0.154 (7)	0.029 (6)	0.035 (6)	0.000 (6)
F4'	0.278 (8)	0.222 (8)	0.255 (8)	0.045 (6)	0.017 (6)	0.031 (6)
F5'	0.218 (8)	0.225 (8)	0.176 (7)	0.096 (7)	−0.003 (6)	0.015 (6)
F6'	0.207 (8)	0.168 (7)	0.105 (5)	0.036 (6)	0.012 (5)	0.052 (4)
O6'	0.217 (10)	0.128 (7)	0.096 (6)	0.019 (7)	−0.006 (6)	−0.010 (5)

### Geometric parameters (Å, º)

**Table:**

C1—F2	1.307 (7)	C21—O4	1.348 (5)
C1—F3'	1.312 (8)	C21—C22	1.464 (6)
C1—F1	1.337 (7)	C22—C27	1.387 (6)
C1—F2'	1.367 (8)	C22—C23	1.391 (7)
C1—F3	1.372 (7)	C23—C24	1.340 (8)
C1—F1'	1.394 (9)	C23—H23	0.9300
C1—C2	1.487 (8)	C24—C25	1.340 (9)
C2—C7	1.366 (7)	C24—H24	0.9300
C2—C3	1.389 (7)	C25—C26	1.383 (8)
C3—C4	1.353 (6)	C25—H25	0.9300
C3—H3	0.9300	C26—C27	1.344 (7)
C4—C5	1.392 (6)	C26—O6'	1.418 (9)
C4—Cl1	1.723 (4)	C26—O6	1.444 (9)
C5—O1	1.355 (5)	C27—H27	0.9300
C5—C6	1.385 (6)	C28—C29	1.3900
C6—C7	1.352 (6)	C28—C33	1.3900
C6—H6	0.9300	C28—O6	1.442 (12)
C7—H7	0.9300	C29—C30	1.3900
C8—C9	1.360 (6)	C29—Cl2	1.770 (9)
C8—C13	1.376 (5)	C30—C31	1.3900
C8—O1	1.409 (5)	C30—H30	0.9300
C9—C10	1.368 (6)	C31—C32	1.3900
C9—H9	0.9300	C31—C34	1.489 (11)
C10—C11	1.382 (6)	C32—C33	1.3900
C10—H10	0.9300	C32—H32	0.9300
C11—C12	1.385 (5)	C33—H33	0.9300
C11—H11	0.9300	C34—F4	1.321 (9)
C12—C13	1.386 (5)	C34—F5	1.330 (9)
C12—C14	1.479 (5)	C34—F6	1.343 (9)
C13—H13	0.9300	C28'—O6'	1.295 (13)
C14—O2	1.195 (4)	C28'—C29'	1.3900
C14—O3	1.356 (4)	C28'—C33'	1.3900
C15—C20	1.363 (5)	C29'—C30'	1.3900
C15—C16	1.384 (6)	C29'—Cl2'	1.673 (7)
C15—O3	1.407 (4)	C30'—C31'	1.3900
C16—C17	1.365 (6)	C30'—H30'	0.9300
C16—H16	0.9300	C31'—C32'	1.3900
C17—C18	1.368 (6)	C31'—C34'	1.517 (10)
C17—H17	0.9300	C32'—C33'	1.3900
C18—C19	1.362 (6)	C32'—H32'	0.9300
C18—H18	0.9300	C33'—H33'	0.9300
C19—C20	1.396 (5)	C34'—F5'	1.327 (8)
C19—O4	1.397 (5)	C34'—F6'	1.341 (8)
C20—H20	0.9300	C34'—F4'	1.351 (9)
C21—O5	1.196 (5)		
			
F2—C1—F3'	117.4 (12)	O5—C21—O4	122.5 (4)
F2—C1—F1	109.1 (6)	O5—C21—C22	124.4 (4)
F3'—C1—F1	51.7 (8)	O4—C21—C22	113.1 (4)
F2—C1—F2'	39.9 (6)	C27—C22—C23	118.1 (4)
F3'—C1—F2'	106.4 (8)	C27—C22—C21	122.6 (4)
F1—C1—F2'	135.0 (10)	C23—C22—C21	119.4 (4)
F2—C1—F3	107.1 (7)	C24—C23—C22	121.0 (5)
F3'—C1—F3	50.8 (8)	C24—C23—H23	119.5
F1—C1—F3	102.4 (6)	C22—C23—H23	119.5
F2'—C1—F3	71.8 (7)	C23—C24—C25	120.9 (6)
F2—C1—F1'	60.3 (7)	C23—C24—H24	119.5
F3'—C1—F1'	103.6 (8)	C25—C24—H24	119.5
F1—C1—F1'	59.1 (8)	C24—C25—C26	119.2 (6)
F2'—C1—F1'	100.0 (7)	C24—C25—H25	120.4
F3—C1—F1'	145.4 (9)	C26—C25—H25	120.4
F2—C1—C2	115.0 (6)	C27—C26—C25	121.2 (6)
F3'—C1—C2	127.5 (11)	C27—C26—O6'	127.3 (7)
F1—C1—C2	113.1 (6)	C25—C26—O6'	108.8 (7)
F2'—C1—C2	110.7 (9)	C27—C26—O6	118.9 (7)
F3—C1—C2	109.3 (6)	C25—C26—O6	117.8 (6)
F1'—C1—C2	105.1 (9)	O6'—C26—O6	32.3 (6)
C7—C2—C3	120.0 (4)	C26—C27—C22	119.5 (5)
C7—C2—C1	120.2 (5)	C26—C27—H27	120.2
C3—C2—C1	119.8 (5)	C22—C27—H27	120.2
C4—C3—C2	119.5 (4)	C5—O1—C8	118.1 (3)
C4—C3—H3	120.3	C14—O3—C15	117.1 (3)
C2—C3—H3	120.3	C21—O4—C19	117.9 (3)
C3—C4—C5	121.2 (4)	C29—C28—C33	120.0
C3—C4—Cl1	119.5 (4)	C29—C28—O6	117.6 (10)
C5—C4—Cl1	119.3 (3)	C33—C28—O6	122.1 (10)
O1—C5—C6	125.3 (4)	C28—C29—C30	120.0
O1—C5—C4	116.8 (4)	C28—C29—Cl2	124.1 (8)
C6—C5—C4	117.9 (4)	C30—C29—Cl2	115.6 (8)
C7—C6—C5	121.1 (4)	C29—C30—C31	120.0
C7—C6—H6	119.4	C29—C30—H30	120.0
C5—C6—H6	119.4	C31—C30—H30	120.0
C6—C7—C2	120.2 (4)	C32—C31—C30	120.0
C6—C7—H7	119.9	C32—C31—C34	121.0 (5)
C2—C7—H7	119.9	C30—C31—C34	118.5 (5)
C9—C8—C13	122.0 (4)	C33—C32—C31	120.0
C9—C8—O1	119.2 (4)	C33—C32—H32	120.0
C13—C8—O1	118.7 (3)	C31—C32—H32	120.0
C8—C9—C10	119.0 (4)	C32—C33—C28	120.0
C8—C9—H9	120.5	C32—C33—H33	120.0
C10—C9—H9	120.5	C28—C33—H33	120.0
C9—C10—C11	121.2 (4)	F4—C34—F5	111.1 (9)
C9—C10—H10	119.4	F4—C34—F6	111.5 (9)
C11—C10—H10	119.4	F5—C34—F6	110.0 (8)
C10—C11—C12	118.9 (4)	F4—C34—C31	106.9 (11)
C10—C11—H11	120.6	F5—C34—C31	106.0 (13)
C12—C11—H11	120.6	F6—C34—C31	111.1 (13)
C11—C12—C13	120.3 (4)	C28—O6—C26	116.3 (10)
C11—C12—C14	122.3 (4)	O6'—C28'—C29'	127.3 (11)
C13—C12—C14	117.4 (3)	O6'—C28'—C33'	112.2 (11)
C8—C13—C12	118.5 (3)	C29'—C28'—C33'	120.0
C8—C13—H13	120.7	C28'—C29'—C30'	120.0
C12—C13—H13	120.7	C28'—C29'—Cl2'	115.6 (8)
O2—C14—O3	122.7 (4)	C30'—C29'—Cl2'	124.4 (8)
O2—C14—C12	124.9 (4)	C29'—C30'—C31'	120.0
O3—C14—C12	112.4 (3)	C29'—C30'—H30'	120.0
C20—C15—C16	122.6 (4)	C31'—C30'—H30'	120.0
C20—C15—O3	116.5 (3)	C32'—C31'—C30'	120.0
C16—C15—O3	120.9 (3)	C32'—C31'—C34'	121.0 (4)
C17—C16—C15	117.1 (4)	C30'—C31'—C34'	119.0 (4)
C17—C16—H16	121.4	C33'—C32'—C31'	120.0
C15—C16—H16	121.4	C33'—C32'—H32'	120.0
C16—C17—C18	122.8 (4)	C31'—C32'—H32'	120.0
C16—C17—H17	118.6	C32'—C33'—C28'	120.0
C18—C17—H17	118.6	C32'—C33'—H33'	120.0
C19—C18—C17	118.4 (4)	C28'—C33'—H33'	120.0
C19—C18—H18	120.8	F5'—C34'—F6'	108.8 (8)
C17—C18—H18	120.8	F5'—C34'—F4'	112.4 (8)
C18—C19—C20	121.5 (4)	F6'—C34'—F4'	106.6 (8)
C18—C19—O4	119.1 (4)	F5'—C34'—C31'	105.2 (7)
C20—C19—O4	119.4 (4)	F6'—C34'—C31'	111.3 (10)
C15—C20—C19	117.6 (4)	F4'—C34'—C31'	112.4 (12)
C15—C20—H20	121.2	C28'—O6'—C26	116.6 (10)
C19—C20—H20	121.2		
			
F2—C1—C2—C7	179.6 (8)	C23—C22—C27—C26	1.7 (8)
F3'—C1—C2—C7	4.4 (15)	C21—C22—C27—C26	−178.0 (5)
F1—C1—C2—C7	−54.1 (9)	C6—C5—O1—C8	−5.4 (5)
F2'—C1—C2—C7	136.4 (9)	C4—C5—O1—C8	174.6 (3)
F3—C1—C2—C7	59.2 (8)	C9—C8—O1—C5	101.9 (4)
F1'—C1—C2—C7	−116.5 (11)	C13—C8—O1—C5	−81.9 (4)
F2—C1—C2—C3	−1.8 (11)	O2—C14—O3—C15	−9.2 (5)
F3'—C1—C2—C3	−177.0 (13)	C12—C14—O3—C15	171.2 (3)
F1—C1—C2—C3	124.4 (7)	C20—C15—O3—C14	−108.2 (4)
F2'—C1—C2—C3	−45.0 (11)	C16—C15—O3—C14	74.5 (4)
F3—C1—C2—C3	−122.2 (7)	O5—C21—O4—C19	6.7 (6)
F1'—C1—C2—C3	62.1 (11)	C22—C21—O4—C19	−173.5 (3)
C7—C2—C3—C4	−1.3 (10)	C18—C19—O4—C21	−119.5 (4)
C1—C2—C3—C4	−179.9 (6)	C20—C19—O4—C21	63.5 (5)
C2—C3—C4—C5	−0.1 (8)	C33—C28—C29—C30	0.0
C2—C3—C4—Cl1	179.9 (5)	O6—C28—C29—C30	174.0 (17)
C3—C4—C5—O1	−179.1 (4)	C33—C28—C29—Cl2	−174.4 (14)
Cl1—C4—C5—O1	0.9 (5)	O6—C28—C29—Cl2	−0.4 (15)
C3—C4—C5—C6	0.9 (7)	C28—C29—C30—C31	0.0
Cl1—C4—C5—C6	−179.1 (3)	Cl2—C29—C30—C31	174.9 (13)
O1—C5—C6—C7	179.8 (4)	C29—C30—C31—C32	0.0
C4—C5—C6—C7	−0.2 (6)	C29—C30—C31—C34	−172 (2)
C5—C6—C7—C2	−1.2 (8)	C30—C31—C32—C33	0.0
C3—C2—C7—C6	2.0 (9)	C34—C31—C32—C33	172 (2)
C1—C2—C7—C6	−179.4 (6)	C31—C32—C33—C28	0.0
C13—C8—C9—C10	−0.4 (6)	C29—C28—C33—C32	0.0
O1—C8—C9—C10	175.6 (4)	O6—C28—C33—C32	−173.7 (17)
C8—C9—C10—C11	1.6 (7)	C32—C31—C34—F4	−14.0 (17)
C9—C10—C11—C12	−0.9 (7)	C30—C31—C34—F4	158.2 (12)
C10—C11—C12—C13	−0.9 (6)	C32—C31—C34—F5	104.6 (14)
C10—C11—C12—C14	−179.2 (4)	C30—C31—C34—F5	−83.2 (15)
C9—C8—C13—C12	−1.4 (6)	C32—C31—C34—F6	−135.9 (12)
O1—C8—C13—C12	−177.4 (3)	C30—C31—C34—F6	36.3 (18)
C11—C12—C13—C8	2.0 (6)	C29—C28—O6—C26	61.6 (15)
C14—C12—C13—C8	−179.6 (3)	C33—C28—O6—C26	−124.6 (12)
C11—C12—C14—O2	172.4 (4)	C27—C26—O6—C28	41.3 (13)
C13—C12—C14—O2	−5.9 (6)	C25—C26—O6—C28	−155.0 (10)
C11—C12—C14—O3	−8.0 (5)	O6'—C26—O6—C28	−73.7 (15)
C13—C12—C14—O3	173.7 (3)	O6'—C28'—C29'—C30'	−170.9 (14)
C20—C15—C16—C17	1.2 (6)	C33'—C28'—C29'—C30'	0.0
O3—C15—C16—C17	178.3 (4)	O6'—C28'—C29'—Cl2'	8.6 (14)
C15—C16—C17—C18	0.1 (7)	C33'—C28'—C29'—Cl2'	179.5 (9)
C16—C17—C18—C19	−0.9 (7)	C28'—C29'—C30'—C31'	0.0
C17—C18—C19—C20	0.4 (6)	Cl2'—C29'—C30'—C31'	−179.4 (10)
C17—C18—C19—O4	−176.5 (4)	C29'—C30'—C31'—C32'	0.0
C16—C15—C20—C19	−1.7 (5)	C29'—C30'—C31'—C34'	−179.2 (15)
O3—C15—C20—C19	−178.9 (3)	C30'—C31'—C32'—C33'	0.0
C18—C19—C20—C15	0.8 (6)	C34'—C31'—C32'—C33'	179.2 (15)
O4—C19—C20—C15	177.7 (3)	C31'—C32'—C33'—C28'	0.0
O5—C21—C22—C27	174.1 (5)	O6'—C28'—C33'—C32'	172.2 (12)
O4—C21—C22—C27	−5.7 (6)	C29'—C28'—C33'—C32'	0.0
O5—C21—C22—C23	−5.6 (7)	C32'—C31'—C34'—F5'	−10.0 (13)
O4—C21—C22—C23	174.6 (4)	C30'—C31'—C34'—F5'	169.1 (9)
C27—C22—C23—C24	0.5 (9)	C32'—C31'—C34'—F6'	107.7 (10)
C21—C22—C23—C24	−179.8 (5)	C30'—C31'—C34'—F6'	−73.1 (12)
C22—C23—C24—C25	−1.8 (11)	C32'—C31'—C34'—F4'	−132.7 (10)
C23—C24—C25—C26	0.9 (12)	C30'—C31'—C34'—F4'	46.5 (14)
C24—C25—C26—C27	1.4 (12)	C29'—C28'—O6'—C26	−88.4 (16)
C24—C25—C26—O6'	164.3 (9)	C33'—C28'—O6'—C26	100.1 (12)
C24—C25—C26—O6	−161.9 (8)	C27—C26—O6'—C28'	−6.7 (19)
C25—C26—C27—C22	−2.6 (10)	C25—C26—O6'—C28'	−168.2 (11)
O6'—C26—C27—C22	−162.2 (9)	O6—C26—O6'—C28'	79.3 (16)
O6—C26—C27—C22	160.4 (6)		

### Hydrogen-bond geometry (Å, º)

**Table:**

*D*—H···*A*	*D*—H	H···*A*	*D*···*A*	*D*—H···*A*
C20—H20···O1^i^	0.93	2.50	3.412 (5)	166
C10—H10···O2^ii^	0.93	2.59	3.152 (5)	120

Symmetry codes: (i) −*x*+2, −*y*+2, −*z*+1; (ii) *x*+1, *y*, *z*.
